# Progression of mild sleep-disordered breathing in children managed with watchful waiting

**DOI:** 10.1007/s44470-026-00082-y

**Published:** 2026-07-07

**Authors:** Erin M. Kirkham, Stacey Ishman, Susan Garetz, Cristina M. Baldassari, Ron B. Mitchell, Christopher Liu, Ignacio E. Tapia, Lisa M. Elden, Fauziya Hassan, Sally Ibrahim, Kristie Ross, Mengqi Cen, Rui Wang, Susan Redline, Ronald D. Chervin

**Affiliations:** 1 Department of Otolaryngology–Head and Neck Surgery, University of Michigan, Ann Arbor, MI USA; 2 Ascension St Vincent-Peyton Manning Children’s Hospital, Indianapolis, IN USA; 3 Department of Otolaryngology, Eastern Virginia Medical School, Children’s Hospital of The King’s Daughters, Norfolk, VA USA; 4 Departments of Otolaryngology-Head and Neck Surgery and Pediatric Sleep Disorders Center, University of Texas Southwestern Medical Center, Dallas, TX USA; 5 Division of Pediatric Pulmonology, Department of Pediatrics, University of Miami Miller School of Medicine, Miami, FL USA; 6 Division of Pediatric Otolaryngology, Perelman School of Medicine, Children’s Hospital of Philadelphia, University of Pennsylvania, Philadelphia, PA USA; 7 Sleep Disorders Center, Department of Pediatrics and Communicable Diseases, University of Michigan, Ann Arbor, MI USA; 8 Department of Pediatrics, University Hospitals Rainbow Babies & Children’s Hospital, Cleveland, OH USA; 9 Department of Population Medicine, Harvard Pilgrim Health Care Institute and Harvard Medical School, Boston, MA USA; 10 Department of Biostatistics, Harvard T.H. Chan School of Public Health, Boston, MA USA; 11 Department of Medicine, Brigham and Women’s Hospital, Harvard Medical School, Boston, MA USA; 12 Sleep Disorders Center, Department of Neurology, University of Michigan, Ann Arbor, MI USA

**Keywords:** Snoring, Sleep-disordered breathing, Sleep apnea, Pediatric, Adenotonsillectomy, PATS

## Abstract

**Purpose:**

To identify clinical characteristics that may be associated with persistence or progression of mild sleep-disordered breathing (SDB) in children who are observed without surgery.

**Methods:**

This is a secondary analysis of the control arm of the Pediatric Adenotonsillectomy Trial for Snoring (PATS), which randomized 458 children aged 3.0 to 12.9 years with mild SDB (snoring with obstructive apnea–hypopnea index [oAHI] < 3 events/hour) to early adenotonsillectomy (eAT) versus watchful waiting with supportive care (WWSC). Participants were assessed at baseline and 12 months with the Pediatric Sleep Questionnaire–Sleep-Related Breathing Disorder (PSQ-SRBD) scale and polysomnography (PSG). We tested for factors predictive of either (1) PSG progression defined by a 12-month oAHI ≥ 3 or (2) symptom persistence or progression defined by a 12-month PSQ-SRBD score ≥ 0.33.

**Results:**

A total of 234 participants were observed (mean age 6.2 years, 111 [47%] female, 65 [28%] Black or African American, 37 [16%] Hispanic). Overall, only 13% (*n* = 20/150) progressed to an oAHI ≥ 3 on repeat PSG, whereas over half (*n* = 110/192, 57%) had symptom persistence/progression. Of the children with persistent or progressive symptoms, 18% (*n* = 16/103) progressed to oAHI ≥ 3. In unadjusted analyses, PSG progression was associated with Black race (OR 2.71, 95% CI 1.03–7.17) and higher baseline PSQ-SRBD (OR 1.74, 95% CI 1.03–3.08). Symptom persistence/progression was predicted by asthma (OR 2.70 [95% CI 1.29, 6.01]), ADHD (OR 3.73 [95% CI 1.13, 16.88]), and tobacco smoke exposure (OR 2.41 [95% CI 1.08–5.82]), while children with larger tonsils (grade III–IV) had lower odds of progression (OR 0.42 [95% CI 0.22–0.78]).

**Conclusions:**

A high symptom burden is common after a year of WWSC for mild SDB, but only a minority of children progressed on PSG. Black race and several clinical characteristics and symptom scores were associated with higher likelihood of progression.

**Trial registration:**

ClinicalTrials.gov Identifier: NCT0256204.

**Brief summary:**

**Current knowledge/study rationale:**

Can clinical information predict persistence or progression of mild sleep-disordered breathing in children who are observed for 1 year without adenotonsillectomy?

**Study impact:**

Polysomnographic progression to obstructive sleep apnea occurs in a minority (13%) of those observed while symptom persistence or progression is more common (57%). Children who are Black or who have asthma, attention-deficit hyperactivity disorder, environmental tobacco exposure, and a high symptom burden could be considered stronger candidates than their peers for earlier adenotonsillectomy for symptomatic mild sleep-disordered breathing.

## Introduction

Obstructive sleep-disordered breathing (SDB) is characterized by upper airway resistance during sleep and ranges in severity from snoring to obstructive sleep apnea (OSA) [1]. Children with SDB experience cognitive impairment [2], academic difficulties [3], behavioral problems [4, 5], and reduced quality of life compared to children without SDB [6, 7]. Present in an estimated 6–17% of children, SDB is associated with significant family and societal impact and increased healthcare cost [8–10]. Adenotonsillectomy (AT) is the first-line treatment for children with SDB, but whether and when to perform surgery is not always clear. The decision is particularly difficult when a child has mild SDB, defined here as frequent snoring without substantial apneas, hypopneas, or gas exchange abnormalities on an overnight polysomnogram (PSG).

Just over 40% of children diagnosed with mild-to-moderate OSA experience spontaneous improvement on PSG within a year without treatment, while only 15% experience substantial improvement in symptoms [11]. Early small studies suggest that most children with primary snoring do not progress to OSA over time [12, 13]. Observation for children whose SDB is likely to resolve may avoid the risks and cost of unnecessary treatment. Conversely, earlier intervention in children whose SDB is likely to persist or progress may reduce the negative health and developmental impact of SDB, which may in turn reduce healthcare utilization and societal burden [6, 14, 15]. Although children with mild SDB may have a higher likelihood of spontaneous resolution than those with more severe SDB [11], persistent SDB—even when it remains mild on PSG—is still consequential. Children with mild SDB are at continued risk for behavioral, quality-of-life, and even cardiovascular morbidity that is reversible with treatment, which argues against observation in all cases [9, 16, 17].

The Pediatric Adenotonsillectomy Trial for Snoring (PATS) was designed to test the impact of early adenotonsillectomy (eAT) versus watchful waiting with supportive care (WWSC) on behavior and cognition in children with mild SDB [18]. Mild SDB was defined as snoring ≥ 3 nights per week with an obstructive apnea–hypopnea index (oAHI) < 3 events per hour. Our objective in this exploratory analysis was to test for baseline clinical variables that may predict either (1) polysomnographic progression of mild SDB to greater-than-mild SDB (oAHI ≥ 3) or (2) symptom persistence or progression defined as a Pediatric Sleep Questionnaire–Sleep-Related Breathing Disorder (PSQ-SRBD) score ≥ 0.33 (range 0–1) among PATS participants who underwent 12 months of watchful waiting [19]. We hypothesized that a set of clinical variables commonly available at baseline may predict progression or persistence of mild SDB in children. Knowledge of clinical predictors of mild SDB progression or persistence may help clinicians identify children most likely to benefit from early AT [11, 20, 21].

## Methods

The study was approved by a central institutional review board (IRB, Children’s Hospital of Philadelphia) and the IRB at each site. An external NIH-appointed data safety monitoring board provided study oversight. Informed consent was obtained in written or electronic format from parents or guardians and assent from children 7 years or older upon enrollment (according to local IRB requirements). The present analysis was exploratory and not prespecified. While the PATS trial was a randomized controlled trial, this is a secondary analysis of the watchful waiting cohort, and therefore, we followed the Strengthening the Reporting of Observational Studies in Epidemiology (STROBE) guidelines for cohort studies.

### PATS study design

The PATS study design and primary outcomes are published previously. PATS was a multicenter, single-blinded randomized controlled trial of AT versus WWSC conducted from 2016 to 2021 [18, 22]. Children aged 3.0 to 12.9 years were recruited from sleep, otolaryngology, and pediatric clinics at seven tertiary referral centers across the United States. Each child underwent a baseline assessment that included a history and physical, validated caregiver-reported symptom and quality-of-life questionnaires, centrally scored PSG, and standardized behavioral and cognitive tests. Children were included if they had mild SDB (defined in PATS to include primary snoring), had tonsillar hypertrophy (defined as Brodsky grade II or greater), and were deemed candidates for AT by an otolaryngologist [18, 22]. Mild SDB was defined in the PATS trial as (1) caregiver-reported snoring ≥ 3 nights per week and (2) PSG that demonstrated an obstructive apnea index < 1 event/hour and oAHI of < 3 events/hour without oxyhemoglobin desaturation < 90%. Children were excluded for recurrent tonsillitis, severe obesity (body mass index [BMI] *z* score ≥ 3), and/or severe chronic health conditions or psychological or behavioral impairment. Participants were randomized 1:1 to eAT or WWSC and followed for 12 months.

### Study procedures

Data were collected at baseline and 12 months later, with caregiver-reported assessments also collected at an interim 6-month visit [11]. Caregivers reported demographic information (age, sex, race, ethnicity, and income) as well as health information (environmental tobacco smoke exposure; diagnosis or treatment for asthma, allergies, and/or ADHD) via standardized questionnaires. Caregiver-reported child race was collected using National Institutes of Health categories. Caregiver-reported child ethnicity was categorized as non-Hispanic or Hispanic. Environmental tobacco smoke exposure was considered positive if the primary caregiver reported currently smoking one or more cigarette(s) per day or if the child’s urinary cotinine assay levels were greater than or equal to 5 ng/mL. Physical exam included height, weight, waist and hip circumference, tonsil size, and palate exam. The body mass index (BMI) percentiles and *z* scores were based on Centers for Disease Control (CDC) growth charts adjusted for age and sex [23]. Tonsil size was assessed at baseline with the Brodsky scale (grades I–IV). As only children with grade II or greater tonsils were included in the study, tonsil size was dichotomized for analysis into grade II versus grade III/IV [24]. The palate position was scored on oral cavity exam both with protrusion of the tongue (Mallampati scale) as well as without (Friedman scale). Both scales were dichotomized into grades I/II (uvula + tonsils/only uvula visible) versus grades III/IV (base of uvula/no uvula visible) [25, 26].

### Sleep questionnaires

Baseline data were analyzed from three caregiver-reported validated questionnaires. The 22-item Pediatric Sleep Questionnaire–Sleep-Related Breathing Disorder (PSQ-SRBD) scale has a score range of 0 to 1; higher scores indicate greater SDB symptom impact and a score ≥ 0.33 indicates a clinically significant symptom impact [19]. The PSQ-SRBD normalized score is the individual score minus group mean divided by the standard deviation. The 8-item Epworth Sleepiness Scale (ESS) modified for children has a score range from 0 to 24; higher scores indicate greater daytime sleepiness; scores ≥ 10 suggest excessive sleepiness in children [27–29]. The 18-item OSA-18 has a score range from 18 to 126; higher scores indicate worse OSA-specific quality of life; a score ≥ 60 indicates significant negative quality-of-life impact [30, 31]. The ESS and OSA-18 scores were not normalized.

### Neurobehavioral assessments

Baseline data were analyzed from three validated, age-appropriate, caregiver-reported assessments of behavior: The Behavior Rating Inventory of Executive Function (BRIEF), the Global Conners Parent Rating Scale 3rd edition short form, and the Childhood Behavior Checklist (CBCL) [32–36]. The total scores scaled to population means (T scores) were used for analysis of each instrument. For all three instruments, a higher T score reflects worse behavior and a T score ≥ 65 is often considered abnormally elevated.

### Polysomnography

All participants underwent full, overnight attended PSG at baseline and were invited for PSG at 12-month follow-up. Certified technologists who were not aware of the child’s clinical presentation or any previous PSG results scored each PSG at a central data coordinating center according to published American Academy of Sleep Medicine recommendations for children [37].

### Biostatistical analysis

**Fig. 1:**
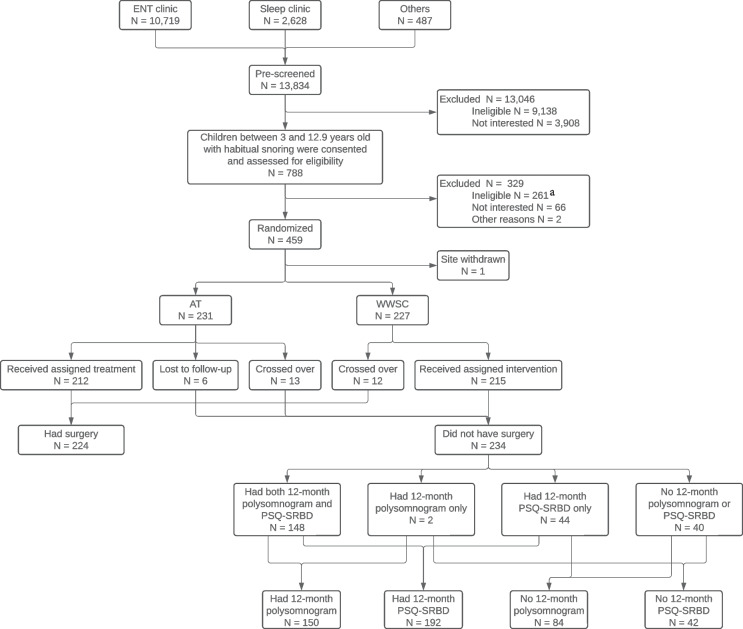
Flow diagram of study participants. ^a^Reasons for exclusion from participation included apnea–hypopnea index out of range; severe, chronic health problems; use of study-restricted medications; no report of habitual snoring; tonsillar size less than II on Brodsky scale; and lack of clinical equipoise

Analysis was performed on data from the 234 (51%) children who did not have surgery. Of those, 215 had been randomized to WWSC and received the assigned intervention, 13 were crossovers who were randomized to eAT but did not undergo surgery, and 6 were randomized to eAT but were lost to follow-up (Fig. 1).

Baseline data were summarized as frequencies for categorical variables and means with standard deviations (SDs) for continuous variables. Data are summarized overall and by groups defined by whether or not they experienced polysomnographic progression of mild SDB to AHI ≥ 3 at 12 months of follow-up and, separately, whether they experienced symptomatic persistence or progression as defined by a PSQ-SRBD score ≥ 0.33 at 12 months. Persistence was defined by both baseline and follow-up PSQ-SRBD score ≥ 0.33; progression was a baseline PSQ-SRBD score < 0.33 with a follow-up score ≥ 0.33. Bivariable comparisons between participants who did and did not experience specified outcomes were assessed with chi-square or Fisher’s exact tests for categorical variables and independent *t*-tests for continuous variables.

Unadjusted measures of association between baseline variables and the 12-month outcomes were tested with bivariable logistic regression models and presented as odds ratios and 95% confidence intervals. We also fit multivariable logistic regression models to attempt to identify the best combined set of variables associated with each outcome. We took two approaches to initial variable selection. The first excluded caregiver-reported sleep-related symptoms and neurobehavioral inventories as these are not routinely administered in the clinical setting. The second approach included these inventories.

We selected predictor variables for multivariable models based on their bivariable association with the outcome (*p* < 0.2). To address multicollinearity between variables, only the variable with the stronger association was retained. Missing data were handled using multiple imputation. For each of 10 imputed datasets, we fit logistic regression models for all possible predictor combinations and calculated log-likelihoods. The pooled Akaike information criterion (AIC) was computed by averaging log-likelihoods across imputations and applying the AIC formula; the model with the lowest pooled AIC was selected [38].

To assess model performance, we performed tenfold multiple imputation, splitting each imputed dataset into training (80%) and testing (20%) subsets. The final model was fit on training sets, and the area under the curve (AUC) was calculated on testing sets using Rubin’s Rules [39]. We estimated standard errors for the pooled AUCs using 500 bootstrap samples [40]. Statistical tests were two-tailed with significance set at *p* < 0.05. Analyses were conducted in R, version 3.6.2 (R Foundation for Statistical Computing).

## Results

Among the 234 children with mild SDB who did not undergo surgery, 150 (64%) were studied with 12-month polysomnography and 192 (82%) had caregiver-reported 12-month PSQ-SRBD scores. At baseline, the sample had an average age of 6.2 (SD 2.4) years, 53% (*n* = 123) were male, 28% (*n* = 65) were Black or African American race, and 37 (16%) were Hispanic ethnicity (Table 1). The sample had a mean (SD) BMI *z* score of 0.43 (1.34) and 47 (20%) had obesity. Baseline characteristics of children with complete follow-up PSG data did not differ significantly from those without follow-up PSG. The same was true of children who did and did not have a follow-up PSQ score.

**Table 1:** Baseline characteristics overall and by polysomnographic progression, defined as 12-month obstructive apnea–hypopnea index (oAHI) ≥ 3 (*N* = 150)

	No progression oAHI^b^ < 3 (*n* = 130)	Progression oAHI ≥ 3 (*n* = 20)	Unadjusted odds ratio for progression	95% CI	*p* value
Demographics, no. (%)^a^					
Age, mean (SD), years	6.2 (2.4)	6 (2.1)	0.98	0.79–1.19	0.83
Sex (male)	69 (53)	13 (65)	1.64	0.63–4.62	0.32
Race					
American Indian/Alaska Native	1 (1)	0 (0)	–	–	0.99
Asian	4 (3)	1 (5)	1.66	0.08–11.97	0.66
Black or African American	35 (27)	10 (50)	2.71	1.03–7.17	0.04
Multiracial	6 (5)	1 (5)	1.09	0.06–6.87	0.94
White	84 (65)	8 (40)	0.37	0.13–0.95	0.04
Ethnicity					
Hispanic/Latinx	14 (11)	1 (5)	0.44	0.02–2.37	0.44
Non-Hispanic/Latinx	116 (89)	19 (95)	2.29	0.42–42.74	0.44
Household income < $30,000/year	31 (26)	6 (32)	1.31	0.43–3.63	0.61
Clinical history, no. (%)					
Asthma	30 (24)	3 (15)	0.56	0.12–1.81	0.38
Allergies	65 (50)	7 (35)	0.54	0.19–1.40	0.22
ADHD	8 (7)	3 (16)	2.41	0.49–9.38	0.23
ETS^c^	21 (18)	6 (35)	2.55	0.80–7.50	0.10
Anthropometric measures, mean (SD)					
BMI^d^ *z* score	0.4 (1.3)	0.6 (1.4)	1.15	0.80–1.70	0.46
Obesity (BMI *z* score > 1.64)	26 (20)	3 (15)	0.71	0.16–2.30	0.60
Waist circumference (cm)	60.2 (11.6)	62.9 (15.3)	1.02	0.98–1.05	0.35
Neck circumference (cm)	27 (2.7)	27.8 (4.1)	1.09	0.93–1.26	0.27
Exam, no. (%)					
Maximum tonsil grade ≥ III	62 (53)	9 (50)	0.87	0.32–2.38	0.79
Mallampati ≥ 3	25 (23)	4 (22)	0.94	0.25–2.89	0.92
Friedman ≥ 3	39 (36)	7 (39)	1.13	0.39–3.10	0.82

*ADHD* attention deficit hyperactivity disorder, *BMI* body mass index, *CI* confidence interval, *cm* centimeters, *ETS* environmental tobacco smoke exposure, *oAHI* obstructive apnea–hypopnea index

^a^Continuous variables presented as mean (standard deviation); categorical characteristics presented as no. (%)

^b^oAHI defined as the average number of obstructive apneas and hypopneas (hypopneas defined by ≥ 3% oxygen desaturation or arousal) per hour of sleep by polysomnography; higher scores indicate more severe OSA

^c^Environmental tobacco exposure defined as caregiver-reported smoking > 1 cigarette per day and/or child urinary cotinine 5 ng/mL or greater

^d^BMI calculated as weight in kilograms divided by the square of height in meters. BMI *z* scores are age- and sex-standardized transformations of BMI that range from positive to negative infinity

The mean baseline oAHI was 0.8 (SD 0.7). Of those with 12-month PSG data, 20 (13%) progressed to an oAHI ≥ 3. Among the 20 children with oAHI progression, 16 (80%) also had a 12-month PSQ-SRBD ≥ 0.33 (symptom persistence or progression). The unadjusted odds ratios for each potential predictor of PSG progression are shown in Table 1. Children of Black race as compared to the others had 2.71 times the odds of PSG progression (95% CI 1.03, 7.17; *p* = 0.04). Odds of PSG progression were also 74% higher when the normalized baseline PSQ-SRBD score was one standard deviation higher (OR 1.74 [95% CI 1.03, 3.08; *p* = 0.05], Table 2).

**Table 2:** Baseline sleep and neurobehavioral measures overall and by polysomnographic progression, defined as 12-month obstructive apnea–hypopnea index (oAHI) ≥ 3 (*N* = 150)

	No progression oAHI^b^ < 3 (*n* = 130)	Progression oAHI ≥ 3 (*n* = 20)	Unadjusted odds ratio for progression	95% CI	*p* value
Baseline polysomnography, mean (SD)					
oAHI (events/hour)^a^	0.7 (0.6)	1 (0.8)	1.80	0.90–3.57	0.10
oAHI ≥ 1, no. (%)	40 (31)	10 (50)	2.25	0.86–5.90	0.10
REM AHI^d^ (events/hour)	0.3 (0.4)	0.4 (0.4)	2.38	0.78–6.91	0.11
Minimum O_2_ (%)	91.6 (3)	91.8 (2.3)	1.02	0.87–1.21	0.82
% TST with CO_2_ > 50 mmHg	2.8 (10.4)	0.5 (1.3)	0.88	0.53–1.01	0.39
Arousal index^e^ (events/hour)	5.5 (1.8)	5.4 (2)	0.97	0.74–1.26	0.83
Sleep efficiency^f^ (%)	88.5 (7.6)	90.8 (6.2)	1.05	0.98–1.15	0.21
REM sleep (%)	17 (4.7)	17.4 (4.5)	1.02	0.92–1.13	0.72
PLMI^g^ (events/hour)	4.1 (6)	4.6 (4.9)	1.01	0.93–1.08	0.74
Baseline sleep inventories, mean (SD)					
mESS^h^	6.7 (4.7)	8.8 (4.1)	1.10	1.00–1.21	0.05
OSA-18^i^	51.4 (15.8)	57.3 (17.7)	1.02	0.99–1.05	0.13
PSQ-SRBD^c^	0.4 (0.2)	0.5 (0.2)	–	–	0.05
PSQ-SRBD ≥ 0.33, no. (%)	94 (72)	18 (90)	3.45	0.93–22.4	0.11
PSQ-SRBD (normalized)	− 0.1 (0.9)	0.4 (0.8)	1.74	1.03–3.08	0.05
Baseline neurobehavioral inventories, mean (SD)					
BRIEF^j^ GEC T-score	55.3 (11.4)	59.1 (11.3)	1.03	0.99–1.07	0.16
CBCL^k^	52.3 (11.3)	52.8 (12.3)	1.00	0.96–1.05	0.86

*BRIEF GEC* Behavior Rating Inventory of Executive Function Global Executive Composite, *CBCL* Child Behavior Checklist, *CI* confidence interval, *mESS* Modified Epworth Sleepiness Scale, *oAHI* obstructive apnea–hypopnea index, *OSA* obstructive sleep apnea, *PLMI* periodic limb movement index, *PSQ* Pediatric Sleep Questionnaire–Sleep-Related Breathing Disorder scale, *REM* rapid eye movement, *TST* total sleep time

^a^Continuous variables presented as mean (standard deviation); categorical characteristics presented as no. (%)

^b^oAHI defined as the average number of obstructive apneas and hypopneas (hypopneas defined by ≥ 3% oxygen desaturation or arousal) per hour of sleep by polysomnography; higher scores indicate more severe OSA

^c^The PSQ is a symptom inventory with a score range from 0 to 1; higher scores indicate greater severity. A score of 0.33 or greater suggests a high risk for a pediatric sleep-related breathing disorder. PSQ (normalized) is the individual score minus group mean divided by the standard deviation

^d^REM AHI is the number of apneas and hypopneas per hour of rapid eye movement sleep on polysomnography

^e^Arousal index is the number of cortical arousals per hour of sleep on polysomnography

^f^Sleep efficiency is the percentage of time in bed spent asleep

^g^Periodic Limb Movement Index is the number of limb movements per hour of sleep by polysomnography

^h^The Epworth Sleepiness Scale modified for children has a score range of 0 (less) to 24 (more) sleepy. A score of 10 or greater represents excessive daytime sleepiness

^i^The OSA-18 is an OSA-specific quality-of-life instrument with a score range of 18 (best) to 126 (worst). A score of 60 or greater represents a moderate to severe negative effect

^j^The BRIEF GEC includes measures of behavioral regulation, emotion regulation, and cognitive regulation (BRIEF-2, for children ages 5 to 18 years) or inhibitory self-control, flexibility, and emergent metacognition (BRIEF-P, for preschool-aged children). Higher scores indicate worse functioning

^k^The CBCL total problems summary scale comprises internalizing, externalizing, social, thought, and attention problems. Higher scores indicate greater behavioral problems

Among participants with 12-month PSQ-SRBD scores, over half (*n* = 110 or 57%) had a high symptom impact (PSQ-SRBD ≥ 0.33) at follow-up, which had persisted from baseline PSQ-SRBD ≥ 0.33 in the majority (94%, *n* = 103/110) and progressed from a baseline PSQ-SRBD score < 0.33 in the minority (6%, *n* = 7/110). For the seven children that had symptom progression, four had an increase in oAHI, two had a decrease, and one did not have a follow-up PSG. Of those with a baseline PSQ-SRBD score ≥ 0.33, nearly three quarters (74%, *n* = 103/139) still had a PSQ-SRBD score ≥ 0.33 at 12 months. Of the children with persistent or progressive symptoms who also had 12-month PSG, 16 (18%) progressed to oAHI ≥ 3.

On unadjusted analysis, children with asthma (OR 2.70 [95% CI 1.29, 6.01; *p* = 0.01]), ADHD (OR 3.73 [95% CI 1.13, 16.88; *p* = 0.05]), and environmental tobacco smoke exposure (OR 2.41 [95% CI 1.08–5.82]; *p* = 0.04) had higher odds of symptom persistence or progression compared to children without these conditions (Table 3). Children with higher baseline modified Epworth sleepiness scores and worse baseline OSA-related quality of life had higher odds of 12-month PSQ-SRBD ≥ 0.33, as did those with greater baseline behavioral impairments on the BRIEF and CBCL (Table 4).

**Table 3:** Baseline characteristics by symptom persistence or progression, defined as 12-month Pediatric Sleep Questionnaire–Sleep-Related Breathing Disorder score ≥ 0.33^a^ (*N* = 192)

	No persistence or progression PSQ^b^ < 0.33 (*n* = 82)	Persistence or progression PSQ ≥ 0.33 (*n* = 110)	Unadjusted odds ratio for persistence or progression	95% CI	*p* value
Demographics, no. (%)^a^					
Age, mean (SD), years	6.2 (2.5)	6.2 (2.2)	0.99	0.87–1.12	0.81
Sex (male)	43 (52)	58 (53)	1.01	0.57–1.79	0.97
Race					
American Indian/Alaska Native	0 (0)	1 (1)	–	–	0.99
Asian	3 (4)	3 (3)	0.74	0.13–4.08	0.72
Black or African American	20 (24)	33 (30)	1.33	0.70–2.57	0.39
Multiracial	4 (5)	5 (5)	0.93	0.24–3.86	0.91
White	55 (67)	68 (62)	0.79	0.43–1.44	0.45
Ethnicity					
Hispanic/Latinx	14 (17)	11 (10)	0.54	0.23–1.26	0.15
Non-Hispanic/Latinx	68 (83)	99 (90)	1.85	0.80–4.41	0.15
Household income < $30,000/year	16 (21)	29 (29)	1.61	0.81–3.29	0.19
Clinical history, no. (%)					
Asthma	11 (14)	31 (30)	2.70	1.29–6.01	0.01
Allergies	34 (41)	61 (55)	1.76	0.99–3.15	0.06
ADHD	3 (4)	12 (14)	3.73	1.13–16.9	0.05
ETS^c^	9 (12)	24 (25)	2.41	1.08–5.82	0.04
Anthropometric measures, mean (SD)					
BMI^d^ *z* score	0.4 (1.3)	0.5 (1.3)	1.06	0.85–1.32	0.63
Obesity (BMI *z* score > 1.64)	15 (18)	28 (25)	1.53	0.76–3.15	0.24
Waist circumference (cm)	60.1 (11.9)	62.1 (13.1)	1.01	0.99–1.04	0.29
Neck circumference (cm)	27 (2.9)	27.4 (3.3)	1.04	0.95–1.15	0.41
Exam, no. (%)					
Maximum tonsil grade ≥ III	47 (65)	45 (44)	0.42	0.22–0.78	0.01
Mallampati ≥ 3	18 (26)	22 (23)	0.85	0.42–1.76	0.66
Friedman ≥ 3	29 (42)	30 (31)	0.63	0.33–1.19	0.15

*ADHD* attention deficit hyperactivity disorder, *BMI* body mass index, *CI* confidence interval, *cm* centimeters, *ETS* environmental tobacco smoke exposure, *oAHI* obstructive apnea–hypopnea index

^a^Continuous variables presented as mean (standard deviation); categorical characteristics presented as no. (%)

^b^The PSQ is a symptom inventory with a score range from 0 to 1; higher scores indicate greater severity. A score of 0.33 or greater suggests a high risk for a pediatric sleep-related breathing disorder. PSQ (normalized) is the individual score minus group mean divided by the standard deviation

^c^Environmental tobacco exposure defined as caregiver-reported smoking > 1 cigarette per day and/or child urinary cotinine 5 ng/mL or greater

^d^BMI calculated as weight in kilograms divided by the square of height in meters. BMI *z* scores are age- and sex-standardized transformations of BMI that range from positive to negative infinity

**Table 4:** Baseline sleep and neurobehavioral measures by symptom persistence or progression, defined as 12-month Pediatric Sleep Questionnaire–Sleep-Related Breathing Disorder score ≥ 0.33^a^ (*N* = 192)

	No persistence or progression PSQ^c^ < 0.33 (*n* = 82)	Persistence or progression PSQ ≥ 0.33 (*n* = 110)	Unadjusted odds ratio for persistence or progression	95% CI	*p* value
Baseline polysomnography, mean (SD)					
oAHI (events/hour)^b^	0.8 (0.7)	0.8 (0.7)	0.93	0.62–1.39	0.72
oAHI ≥ 1, no. (%)	27 (33)	37 (34)	1.03	0.56–1.90	0.92
REM AHI^d^ (events/hour)	0.4 (0.5)	0.3 (0.4)	0.69	0.34–1.40	0.31
Minimum O_2_ (%)	91.4 (3)	92.2 (2.7)	1.11	1.00–1.24	0.04
% TST with CO_2_ > 50 mmHg	1.6 (5.2)	3.2 (12.4)	1.02	0.99–1.07	0.33
Arousal index^e^ (events/hour)	5.7 (2)	5.6 (1.9)	0.97	0.83–1.12	0.67
Sleep efficiency^f^ (%)	88.4 (7.5)	88.6 (7.3)	1.01	0.97–1.05	0.79
REM sleep (%)	17.3 (3.8)	16.7 (5.1)	0.97	0.91–1.03	0.36
PLMI^g^ (events/hour)	4.1 (4.9)	4.1 (5.9)	1.00	0.95–1.05	0.96
Baseline sleep inventories, mean (SD)					
mESS^h^	5.6 (4.5)	7.6 (4.4)	1.11	1.04–1.20	< 0.01
OSA-18^ i^	43.5 (13.8)	58.5 (16.7)	1.07	1.04–1.10	< 0.01
PSQ-SRBD	0.3 (0.1)	0.5 (0.2)	–	–	< 0.01
PSQ-SRBD ≥ 0.33, no. (%)	36 (44)	103 (94)	18.8	8.24–49.04	< 0.01
PSQ-SRBD (normalized)	− 0.6 (0.8)	0.5 (0.8)	5.88	3.64–10.24	< 0.01
Baseline neurobehavioral inventories, mean (SD)					
BRIEF^j^ GEC T-score	52.1 (11.7)	58.7 (10.3)	1.06	1.03–1.09	< 0.01
CBCL^k^	48.8 (11.2)	55.7 (10.5)	1.06	1.03–1.09	< 0.01

*ADHD* attention deficit hyperactivity disorder, *AHI* apnea–hypopnea index, *BMI* body mass index, *BRIEF GEC* Behavior Rating Inventory of Executive Function Global Executive Composite, *CBCL* Child Behavior Checklist, *cm* centimeters, *ETS* environmental tobacco smoke exposure, *mESS* Modified Epworth Sleepiness Scale, *oAHI* obstructive apnea–hypopnea index, *OSA* obstructive sleep apnea, *PLMI* periodic limb movement index, *PSQ* Pediatric Sleep Questionnaire–Sleep-Related Breathing Disorder scale

^a^Continuous variables presented as mean (standard deviation); categorical characteristics presented as no. (%)

^b^oAHI defined as the average number of obstructive apneas and hypopneas (hypopneas defined by ≥ 3% oxygen desaturation or arousal) per hour of sleep by polysomnography; higher scores indicate more severe OSA

^c^The PSQ is a symptom inventory with a score range from 0 to 1; higher scores indicate greater severity. A score of 0.33 or greater suggests a high risk for a pediatric sleep-related breathing disorder. PSQ (normalized) is the individual score minus group mean divided by the standard deviation

^d^REM AHI is the number of apneas and hypopneas per hour of rapid eye movement sleep on polysomnography

^e^Arousal index is the number of cortical arousals per hour of sleep on polysomnography

^f^Sleep efficiency is the percentage of time in bed spent asleep

^g^Periodic Limb Movement Index is the number of limb movements per hour of sleep by polysomnography

^h^The Epworth Sleepiness Scale modified for children has a score range of 0 (less) to 24 (more) sleepy. A score of 10 or greater represents excessive daytime sleepiness

^i^The OSA-18 is an OSA-specific quality-of-life instrument with a score range of 18 (best) to 126 (worst). A score of 60 or greater represents a moderate to severe negative effect

^j^The BRIEF GEC includes measures of behavioral regulation, emotion regulation, and cognitive regulation (BRIEF-2, for children ages 5 to 18 years) or inhibitory self-control, flexibility, and emergent metacognition (BRIEF-P, for preschool-aged children). Higher scores indicate worse functioning

^k^The CBCL total problems summary scale comprises internalizing, externalizing, social, thought, and attention problems. Higher scores indicate greater behavioral problems

Tonsil grade ≥ III at baseline was associated with lower odds of symptom persistence or progression (OR 0.42 [95% CI 0.22–0.78; *p* = 0.01]). Among children with larger tonsils (grade ≥ III) at baseline, 69% (*n* = 77/111) of caregivers reported a high symptom inventory at baseline, and fewer (49% [*n* = 45/92], 20% difference, 95% CI 7%, 34%) reported the same at follow-up (Table 3). In contrast, for children with smaller tonsils (grade II) at baseline, 75% (*n* = 78/104) of caregivers reported a high symptom inventory at baseline and a similar proportion (70%, *n* = 57/82) reported the same at follow-up (5% difference, 95% CI − 7%, 19%).

The multivariable predictive models for polysomnographic progression are shown in Table 5. When only clinical variables were included without caregiver-reported inventories, the final model retained Black race alone, with low discriminant validity (AUC 0.62, 95% CI 0.50, 0.77). When both clinical variables and caregiver-reported inventories were included, the final model retained Black race, AHI in rapid eye movement (REM) sleep, and the normalized PSQ-SRBD score, with slightly higher but still low discriminant validity (AUC 0.70, 95% CI 0.54, 0.85).

**Table 5:** Multiple-predictor models for polysomnographic progression (12-month obstructive apnea–hypopnea index^a^ ≥ 3, *N* = 150)

Model	Odds ratio (95% CI)	*p* value	AUC (95% CI)
Clinical only^b^			
Black race^c^	2.71 (1.03, 7.13)	0.04	0.62 (0.50–0.77)
Clinical plus caregiver-reported inventories			
Black race^c^	2.43 (0.90, 6.56)	0.08	0.70 (0.54–0.85)
PSQ-SRBD^d^ (normalized)	1.79 (0.70, 3.90)	0.10	
REM AHI^e^	1.68 (1.02, 3.15)	0.04	

*AHI* apnea–hypopnea index, *AUC* area under the curve, *CI* confidence interval, *PSQ-SRBD* Pediatric Sleep Questionnaire–Sleep-Related Breathing Disorder scale, *REM* rapid eye movement

^a^Defined as the average number of obstructive apneas and hypopneas (hypopneas defined by ≥ 3% oxygen desaturation or arousal) per hour of sleep by polysomnography; higher scores indicate more severe OSA

^b^The predictors in the multivariable model were selected using pooled Akaike information criterion (AIC) in a multivariable regression framework with multiply imputed data. The model with the lowest pooled AIC is selected as the best subset of variables that showed associations with polysomnographic progression of OSA on bivariate analyses with the exception of caregiver-reported baseline sleep and neurobehavioral inventories. These were excluded from the “clinical only” model as they are not commonly collected in the clinical setting. Clinical only model excluded caregiver-reported baseline sleep and neurobehavioral inventories, as they are not commonly collected in the clinical setting

^c^Comparator group all other (non-Black) races

^d^The PSQ-SRBD is a symptom inventory with a score range from 0 to 1; higher scores indicate greater severity. A score of 0.33 or greater suggests a high risk for a pediatric sleep-related breathing disorder. PSQ-SRBD (normalized) is the individual score minus group mean divided by the standard deviation

^e^REM AHI is the number of apneas and hypopneas per hour of rapid eye movement sleep on polysomnography

The multivariable predictive models for symptom persistence and/or progression are shown in Table 6. The clinical-only model included asthma, ADHD, minimum oxygen saturation, environmental tobacco smoke exposure, and maximum tonsil grade ≥ III, but each variable was associated with a wide confidence interval and the overall model had low discriminant validity (AUC 0.69, 95% CI 0.59, 0.79). The model that included caregiver-reported inventories retained maximum tonsil grade ≥ III and the normalized PSQ-SRBD score, with good discriminant validity (AUC 0.85, 95% CI 0.79, 0.92).

**Table 6:** Multiple-predictor models for progression or persistence of symptoms (12-month Pediatric Sleep Questionnaire–Sleep-Related Breathing Disorder score^a^ ≥ 0.33, *N* = 192)

Model	Odds ratio (95% CI)	*p* value	AUC (95% CI)
Clinical only^b^			
Asthma	2.10 (0.79, 5.54)	0.13	0.69 (0.59–0.79)
ADHD	2.40 (0.59, 9.83)	0.22	
Minimum O_2_ saturation	1.04 (0.90, 1.19)	0.59	
Environmental tobacco smoke exposure^c^	1.91 (0.68, 5.32)	0.22	
Maximum tonsil grade ≥ III	0.48 (0.22, 1.03)	0.06	
Clinical plus caregiver-reported inventories			
Maximum tonsil grade ≥ III	0.44 (0.20, 0.94)	0.03	0.85 (0.79–0.92)
PSQ-SRBD^c^ (normalized)	5.38 (3.15, 9.18)	< 0.01	

*ADHD* attention deficit hyperactivity disorder, *AUC* area under the curve, *CI* confidence interval, *PSQ-SRBD* Pediatric Sleep Questionnaire–Sleep-Related Breathing Disorder

^a^The Pediatric Sleep Questionnaire–Sleep-Related Breathing Disorder scale is a symptom inventory with a score range from 0 to 1; higher scores indicate greater severity. A score of 0.33 or greater suggests a high risk for a pediatric sleep-related breathing disorder

^b^Clinical only model excluded caregiver-reported baseline sleep and neurobehavioral inventories, as they are not commonly collected in the clinical setting

^c^Environmental tobacco exposure defined as caregiver-reported smoking > 1 cigarette per day and/or child urinary cotinine 5 ng/mL or greater

## Discussion

In this diverse sample of children randomized to defer AT for mild SDB (oAHI < 3), only a minority (13%) progressed to oAHI ≥ 3 on PSG 12 months later. However, a high symptom inventory (PSQ-SRBD score ≥ 0.33) was common at baseline (57%) and 74% of these affected children had symptom persistence at 12 months. Among those without a high symptom inventory at baseline, few (13%) progressed to a high symptom inventory at follow-up. Among those with persistence or progression of symptoms, 18% who underwent repeat PSG had progressed at 12 months. Baseline variables associated with polysomnographic progression over 12 months to oAHI ≥ 3 in either univariable or multivariable models include Black race, higher baseline PSQ-SRBD score, and higher AHI in REM sleep.

On unadjusted analysis, asthma, ADHD, environmental tobacco smoke exposure, and smaller tonsils were each associated with symptom persistence or progression. Worse impairment on the mESS, OSA-18, BRIEF, and CBCL, as well as baseline PSQ-SRBD, was also associated with 12-month PSQ-SRBD ≥ 0.33. The best multivariable predictive model for symptom persistence or progression included baseline PSQ-SRBD scale and tonsillar grade > III, which surprisingly had a protective effect.

PATS was designed as a follow-up to the Childhood Adenotonsillectomy Trial (CHAT), which randomized 5- to 9-year-old children with mild-to-moderate OSA to eAT versus 7 months of WWSC. A substantial proportion (*n* = 82/194, 42%) of the non-surgical group in CHAT (all of whom had OSA [AHI ≥ 5] at baseline) experienced polysomnographic resolution by 7 months of follow up [11]. A higher baseline AHI and a waist circumference > 90th percentile independently predicted PSG persistence of OSA in CHAT. The majority (85%) of observed children who had a high baseline symptom inventory on the PSQ-SRBD scale (score > 0.33) still had a high symptom inventory at follow-up, whether or not they still had OSA on repeat PSG. Only a higher baseline PSQ-SRBD score independently predicted a high score at follow-up. Similar to results from CHAT, we found that among our cohort with mild SDB in PATS, persistent or progressive symptoms were likely to persist with WWSC. Interestingly, persistence or progression of symptoms did not necessarily coincide with progression on PSG in PATS, as 80% of children with a high PSQ-SRBD score (≥ 0.33) at follow-up still had a low oAHI (< 3) on repeat sleep study.

Only limited data are available from community-based observational studies on the progression of untreated SDB in children. In the Penn State Child Cohort, among 89 elementary school children (mean age 8.5 years) with primary snoring, 23 (25.8%) progressed to mild OSA (AHI 2 to < 5 events/h) and 11 (12.4%) to greater than mild OSA (AHI ≥ 5) by adolescence (mean age 16.5 years). For the 45 participants with mild OSA at baseline, 15 (33.3%) persisted, while 6 (13.3%) progressed to AHI ≥ 5 events per hour [41]. Overall, for 134 children in the cohort with either primary snoring or mild OSA (AHI 2 to < 5 events/h) at baseline, 35 (26%) progressed in severity. The higher proportion of patients who progressed compared to that found in the present PATS analysis (13%) may be due to the older age of the cohort at both baseline and follow-up, as well as the difference in cutoffs that defined categories of SDB. Few population-based studies have examined specific risk factors for SDB progression in children [41–43]. One exception was a cohort study conducted in Hong Kong from 2003 to 2005, in which 26 (37%) of 98 children with primary snoring progressed to mild OSA (oAHI 1–5 events/h) while 13 (27%) of 49 with baseline oAHI 1–5 progressed to moderate or greater OSA (oAHI ≥ 5). In the Hong Kong cohort, male sex was a risk factor for PSG progression. As the authors noted, “sex-specific anatomic changes at puberty may explain the male predisposition to pharyngeal collapse and hence higher risk for OSA.” Therefore, the marked differences in age range and follow-up time could explain the lack of a similar finding in PATS, as the Hong Kong cohort was on average 10 years old at baseline and 20 years old at follow-up [44]. While cohort studies consistently report that male sex and obesity are risk factors for incident and prevalent SDB [41–44], we did not find an association between sex or baseline weight status and progression by polysomnography or symptoms (PSQ-SRBD score) in PATS. This may be because PATS included a predominantly prepubertal sample and excluded children with very high BMI.

We found that Black children in comparison with others were more likely to experience PSG progression but not symptomatic persistence/progression. Chervin et al. found the same results among Black versus non-Black children randomized to WWSC in CHAT [11]. Indeed, Black children are more likely than non-Black children to have OSA [45], to be more severely affected [46, 47], and to have persistent OSA whether treated with AT or observed with supportive care [48]. Reasons for this disparity are multifactorial but may relate to chronic exposure to respiratory irritants and allergens common in disadvantaged neighborhoods [49, 50]. Indeed, Black children in PATS were more likely than non-Black children to live in disadvantaged neighborhoods [49]. Further research is needed to improve equity in diagnosis, patient impact, and treatment outcomes of pediatric OSA.

Contrary to expectation, larger baseline tonsil size was associated with lower odds of symptom persistence or progression among observed children. A closer look at our data revealed that a substantial proportion of highly symptomatic children *without* large tonsils are likely to remain symptomatic a year later. This may be due to a “ceiling effect” of tonsil size; those with large tonsils at baseline may be at maximal obstruction whereas those with smaller tonsils have potential for further growth and negative impact on symptoms. Factors apart from tonsillar hypertrophy may contribute to SDB (i.e., adenoid hypertrophy, diffuse inflammation, non-tonsillar sites of obstruction, low pharyngeal tone, and altered respiratory physiology) and these factors may not resolve in 12 months [51–54]. Importantly, PATS did not assess adenoid size at baseline or follow-up. The symptomatic children without tonsillar hypertrophy may have had large adenoids or obstruction at other airway sites that could at least in part account for their persistent symptoms at follow-up. Some data suggest that adenoid size predicts OSA on PSG better than tonsil size in younger children, whereas tonsil size shows inconsistent correlation with OSA on PSG across investigations [55, 56]. A study of 19 children with OSA and obesity found both higher oAHI and higher PSQ scores among participants with ≥ 75% adenoid obstruction on endoscopy [57]. A larger cohort study of 194 children who underwent sleep endoscopy just prior to adenotonsillectomy found that multilevel obstruction in comparison to adenotonsillar obstruction alone was associated with higher PSQ-SRBD scores [58].

Our data also demonstrated that symptoms of SDB improved spontaneously over time in a substantial proportion of children despite baseline large tonsils. This may have been due to a decrease in tonsil size or improved patency of the airway from skeletal growth, decreased inflammation, or weight changes. We can only postulate, as follow-up tonsil size was not recorded as part of the protocol and we did not analyze weight changes as the present analysis was focused only on baseline clinical factors. Our seemingly paradoxical findings on the predictive value of tonsil size for SDB progression or persistence, however, are consistent with the preponderance of available research, which suggests that tonsil size does not correlate well with objective or self-reported OSAS severity [59, 60] or higher odds of improvement after AT [51, 61].

Asthma at baseline was a predictor of symptomatic persistence or progression as measured by the PSQ-SRBD. This is not surprising, as there is an established bidirectional relationship between asthma and SDB in children [62]. Children with asthma in comparison to their peers are twice as likely to have SDB [63], and the prevalence of SDB symptoms among children with asthma is as high as 35% [64]. In addition, severity of asthma correlates with severity of SDB [65, 66], and SDB negatively affects asthma control [67]. A separate analysis of the treatment effect of eAT on asthma symptoms in PATS found a relative reduction in asthma symptoms in the eAT group at 6 and 12 months, though the effect did not reach statistical significance likely due to lack of statistical power [68].

Multiple mechanisms exist by which asthma may contribute to SDB. Asthma is associated with poor sleep quality, poor sleep efficiency, frequent awakenings, and REM sleep-related breathing abnormalities, all of which may contribute to and overlap with nighttime symptoms of SDB [69–71]. Asthma may also contribute to daytime sleepiness and affect cognition [72, 73]. Both arms of PATS received referral for asthma management as part of supportive care throughout the trial. However, in the eAT group, 39% of children with asthma had high symptom inventory on the PSQ-SRBD at follow-up compared to 74% in the WWSC group. These results suggest that asthma management without eAT did not have a substantial impact on SDB symptoms over 12 months of WWSC and provide support for early AT rather than WWSC in children with asthma and mild SDB.

### Strengths and limitations

This study has notable strengths. Patients comprise a national sample of children across a wide age range with clearly defined mild SDB. The data were collected as part of a comparatively large, diverse, multisite randomized clinical trial with a high follow-up rate and a standardized protocol for data collection and integrity. The present analysis also has limitations. The PSQ-SRBD—although well-validated and widely used—may not perfectly capture persistence or progression of the symptoms that characterize mild SDB [74, 75]. Our finding that 57% of participants had persistent or progressive symptoms while only 13% had oAHI progression may partly reflect discrepancies between subjective and objective measures. However, most treatment-related behavioral changes in children with OSA are mediated statistically by the changes in parent-reported sleep-disordered breathing severity on the PSQ-SRBD rather than changes in polysomnographic parameters [76]. It is notable that in this study 80% of children with oAHI progression had high PSQ-SRBD scores at follow-up, which suggests reasonable concordance for the most severe cases. Furthermore, this was an exploratory analysis that was not powered to specifically identify risk factors for persistence or progression of mild SDB. Although this is one of the largest controlled trials of adenotonsillectomy to date, numbers are still modest in size for development of a predictive equation for a relatively rare outcome. Due to the low numbers of children who progressed on PSG, we could not assess potential risk factors for a composite outcome of simultaneous progression on both PSG and symptom measures. In this study, we were specifically interested in baseline characteristics that could assist the clinician in counseling families on treatment options when surgery is under consideration. Therefore, we did not include measures of follow-up change in weight or tonsil size. Additionally, tonsil size was evaluated on clinical exam but adenoid size was not assessed in the WWSC group, which prevented adjustment for adenoid size in analyses. Although follow-up was incomplete for both endpoints (64% with PSG and 82% with PSQ), baseline characteristics did not differ between those who did and did not have complete follow-up data, which decreases the likelihood of selection bias by attrition.

### Clinical implications

Black race and high baseline symptom inventory were associated with PSG progression. However, the relatively low numbers of patients who progressed to oAHI ≥ 3 limited our ability to develop a strong predictive model. Persistence or progression of symptoms was most strongly predicted by a high symptom inventory at baseline and tonsil grade. Symptoms commonly persist or worsen in snoring children with mild SDB even when oAHI does not increase over 12 months. A repeat polysomnogram a year from initial assessment captures only a small group of patients who may progress to OSA and may not always help in clinical decision-making. In the symptomatic snoring child, it may be most valuable to repeat symptom assessment and consider impacts on behavior and quality of life. Our study provides important data on potential predictors of progression that could inform the design of future, larger, and longer-term studies of children. Future research is needed to more clearly identify which children may benefit from earlier adenotonsillectomy versus observation.

## Conclusions

Progression of mild SDB on PSG occurred in a minority of PATS participants and was associated with Black race and a higher baseline PSQ-SRBD symptom inventory score; however, the final prediction model only had modest predictive value. Multiple individual baseline variables each predicted symptomatic progression—and these included asthma, environmental tobacco smoke, higher self- or parent-reported sleepiness ratings, lower OSA-related quality of life, greater behavioral problems, and smaller tonsils. Of all variables examined, the baseline PSQ-SRBD was most strongly associated with follow-up symptoms.

## Data Availability

De-identified data (including data dictionaries) are available upon request via the National Sleep Research Resource for participants for whom there are no regulatory restrictions for data sharing (sleepdata.org).
